# Prostatic artery embolization performed in anteroposterior projections versus steep oblique projections: single centre retrospective comparative analysis

**DOI:** 10.1186/s42155-021-00209-7

**Published:** 2021-02-09

**Authors:** Hippocrates Moschouris, Andreas Dimakis, Marina G. Papadaki, Athanasios Liarakos, Konstantinos Stamatiou, Ioulita Isaakidou, Ilianna Tsetsou, Vasiliki Mylonakou, Katerina Malagari

**Affiliations:** 1 Radiology Department, General Hospital “Tzanio”, Zanni & Afentouli 1 Str., 18536 Piraeus, Greece; 2 Department of Surgery, General Hospital “Tzanio”, Zanni & Afentouli 1 Str., 18536 Piraeus, Greece; 3 Urology Department, General Hospital “Tzanio”, Zanni & Afentouli 1 Str., 18536 Piraeus, Greece; 4 2nd Department of Radiology, University of Athens, “Attikon” Hospital, Rimini 1 Str., Chaidari, 12462 Athens, Greece

**Keywords:** Prostatic artery embolization, Anteroposterior projections, Oblique projections, Pelvic angiography, Dose area product

## Abstract

**Background:**

To present and evaluate an approach for reduction of utilization of steep oblique angiographic projections during prostatic artery embolization (PAE).

**Methods:**

Single-center, retrospective study of patients who underwent bilateral PAE (from October 2018 to November 2019) and in whom it was possible to embolize PA of at least one pelvic side utilizing anteroposterior projections only (AP-PAE group), with the following techniques: Identification of the origin of PA on anteroposterior angiographic views. Utilization of anatomic landmarks from the planning computed tomographic angiography. Distal advancement of the angiographic catheter or microcatheter in the anterior division of internal iliac artery. Gentle probing with microguidewire at the expected site of origin of the PA. The AP-PAE approach was initially applied to all PAE patients during the study period and when this approach failed, additional steep oblique projections were acquired; patients who underwent bilateral PAE, with both anteroposterior and oblique projections for both pelvic sides, formed the standard PAE (S-PAE) group. The AP-PAE group was compared with S-PAE group in terms of baseline clinical and anatomic features, technical/procedural aspects and outcomes.

**Results:**

Forty-six patients (92 pelvic sides) were studied. AP-PAE was feasible in 12/46 patients (26.0%): unilateral AP-PAE in 9/46 patients (19.5%); bilateral AP-PAE in 3/46 patients (6.5%). AP-PAE group had larger prostates (*p* = 0.047) and larger PAs (*p* < 0.001). Body mass index (BMI) and other baseline features were comparable between the two groups (mean BMI, AP-PAE group: 27.9 ± 3.6, S-PAE group: 27.0 ± 3.5, *p* = 0.451). Mean fluoroscopy time and dose area product were lower in AP-PAE group (46.3 vs 57.9 min, *p* = 0.084 and 22,924.9 vs 35,800.4 μGy^.^m^2^, *p* = 0.018, respectively). Three months post PAE, comparable clinical success rates (11/12 vs 31/34, *p* = 0.959) and mean International Prostate Symptom Score reduction (60.2% vs 58.1%, *p* = 0.740) were observed for AP-PAE and for S-PAE group, respectively. No major complications were encountered.

**Conclusion:**

AP-PAE is associated with significant reduction in radiation exposure and appears to be feasible, safe and effective, but it can be applied in a relatively small percentage of patients.

**Supplementary Information:**

The online version contains supplementary material available at 10.1186/s42155-021-00209-7.

## Introduction

Prostatic artery embolization (PAE) is a technically challenging endovascular procedure due to the variant pelvic arterial anatomy, frequent tortuosity and atheromatosis of these arteries, small size of prostatic arteries (PAs) and frequent anastomoses and overlap with arteries of neighbouring organs (Bilhim et al. 2012a, 2012b; Carnevale et al. 2017). To facilitate identification and catheterization of PAs, steep oblique fluoroscopic and angiographic views (ipsilateral oblique at 35^o^-45^o^ with additional caudal-cranial angulation of approximately 10^o^) are routinely utilized as a standard step of the PAE procedure (Bilhim et al. 2012a, 2012b, Carnevale et al. 2017). Thanks to these projections, most of the pelvic arterial branches relevant to PAE (Pudendal, Rectal, Obturator, Vesical Inferior and Superior, under the ipsilateral Oblique view-“PROVISO” acronym, Carnevale et al. 2017) can be identified with relative ease and with limited superposition of other pelvic arteries.

 However, compared to anteroposterior, steep oblique views are associated with a significantly increased radiation dose to patient and staff. Of note, pelvic endovascular procedures are accompanied by significantly higher effective doses for the operator than peripheral vascular interventions and this difference is largely attributable to the frequency of oblique projections during the former (Ingwersen et al. 2013). In a recent systematic review focusing on radiation exposure during PAE (Zumstein et al. 2020), wide variations in dose-area product (DAP) between different PAE studies were found (mean DAP: 3316–86,340 μGy^.^m^2^) and the large potential to reduce this exposure was emphasized. Optimization (meaning that imaging should be performed using doses that are as low as reasonably achievable - “ALARA”) is one of the three main principles of radiation protection (Hertault et al. 2015; Do 2016); it would probably be worth investigating how and if reduction of oblique views could facilitate the implementation of the ALARA principle in the context of PAE.

In practice, in some cases of PAE, PA of at least one pelvic side can be selected and embolized using exclusively anteroposterior fluoroscopic and angiographic imaging. In this report, a series of such cases is reviewed and the relevant technical steps are described. The feasibility, efficacy, safety and potential benefits of this approach (PAE with anteroposterior angiographic imaging only) are evaluated and compared with standard technique of PAE.

## Materials and methods

Institutional review board approval was obtained for this study. 

### Patients, equipment and consent

Patients who were treated with bilateral PAE for symptomatic benign prostatic hyperplasia at a single institution during (approximately) a 1-year period, from October 2018 to November 2019, were retrospectively reviewed. Pre-procedural evaluation, inclusion and exclusion criteria were the same with previous work (Moschouris et al. 2019) and with other PAE studies (Pisco et al. 2016). To eliminate the effects of the learning curve, patients treated during the first 2 years of the local PAE practice (July 2016–September 2018) were excluded. Also excluded were cases of unilateral or failed PAE (*n* = 6 and *n* = 2, respectively) during the study period and procedures which were performed after November 2019, with newer equipment (Axiom Artis Zee, Siemens Healthineers, Erlangen, Germany, instead of Axiom Artis MP, Siemens, which was utilized for all the patients of this study).

Written informed consent was obtained from all patients prior to treatment.

### Technique

**Fig. 1:**
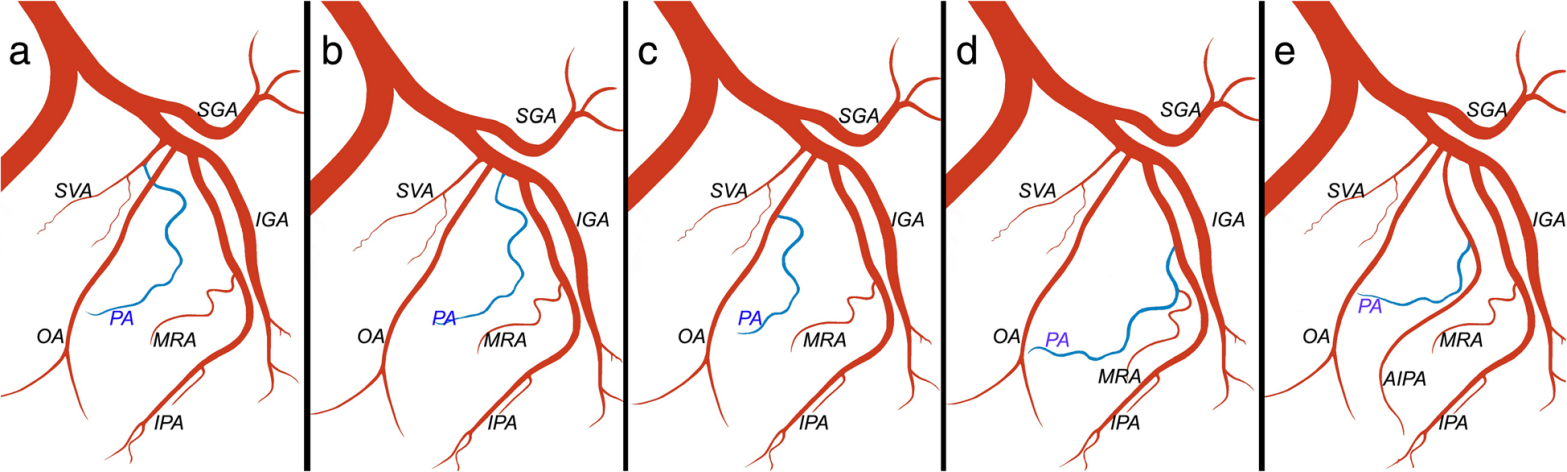
Schematic diagram (ipsilateral oblique view) of the 5 types of prostatic artery (PA, in blue) origin according to de Assis and Carnevale (de Assis et al. 2015). **a** Type I, common origin of the SVA and PA. **b** Type II, PA origin from the anterior division of the internal iliac, independent from and inferior to the SVA. **c**: Type III, PA origin from OA. **d** Type IV, PA origin from IPA. **e** Type V, less common origins, the most frequent of which (presented here) is PA origin from AIPA SVA-superior vesical artery, OA-obturator artery, MRA-middle rectal artery, IPA-internal pudendal artery, AIPA-accessory internal pudendal artery, IGA-inferior gluteal artery, SGA-superior gluteal artery

Pre-procedural clinical and imaging evaluation was performed according to the institute’s standard protocol (Moschouris et al. 2019). Computed-Tomographic Angiography (CTA) was performed for treatment planning to all patients prior to PAE (Table 1 of supplementary material). The origin and course of the PAs was studied on axial CT slices and on Maximum Intensity Projections (MIPs) on coronal, sagittal and oblique sagittal planes. Anatomic landmarks that facilitated localization of the origin of PAs on anteroposterior MIP projections were noted, for subsequent correlation with anteroposterior pelvic digital subtraction angiography (DSA). For each pelvic side, the type of origin of PA was defined according to the classification system (Fig.1), proposed by de Assis et al. (2015). The diameter of the PA was measured at its origin. The tortuosity of the pelvic arteries was assessed and graded, using a practical categorization of a previous PAE study (Enderlein et al. 2020): Grade 1 (mild: kinking < 30 ^o^ in both pelvic sides), Grade 2 (moderate: maximum kinking 30 ^o^- 60^o^ in at least 1 pelvic side), and Grade 3 (severe: multiple kinking 30 ^o^- 60 ^o^ in both sides and kinking of > 60^o^ in at least 1 pelvic side). Angles were measured in coronal MIP images. Finally, in line with a previous report (Hacking et al. 2019), the diameter of the internal iliac arteries was visually assessed and atheromatous stenoses were classified as: Grade 0, 1, 2 and 3 (for no, mild, moderate and severe stenoses, respectively).

With the exception of patients with indwelling bladder catheter (IBC), PAE was performed without previous bladder catheterization. Vascular access was obtained via the right common femoral artery or left radial artery. The latter access (preceded by the Barbeau test and preferred when preprocedural CTA indicated severe tortuosity and/or significant stenoses of the external iliac and femoral arteries) entailed ultrasonographically guided cannulation of the radial artery 1–2 cm proximal to the radial styloid process and utilization of an appropriate introducer (Prelude radial sheath introducer, Merit Medical, South Jordan, UT, USA). The internal iliac arteries (IIAs) were catheterized with a 5 French (Fr.) angiographic catheter. In case of femoral access, 65–80 cm double angle or reverse curve catheters (Cobra1, Simmons 1, Contra 2 or Uterine Artery catheter, Merit Medical, or Boston Scientific, MA, USA,) were utilized for the ipsilateral IIA. If catheterization of the contralateral IIA could not be achieved with these catheters, they were exchanged for a hydrophilic one (Vertebral Glidecath, Terumo Corporation, Japan). In case of left radial access, an 125 cm Multipurpose or Vertebral catheter (Merit Medical) was utilized.

Anteroposterior DSA of the IIAs was performed with manual injection of 10-20 ml of contrast through the angiographic catheter. One or more of the following techniques were applied, in order to perform PAE with anteroposterior imaging only (AP-PAE): 1) Identification of the origin of PA on DSA and subsequent attempt for catheterization, usually with the aid of a roadmap. 2) Utilization of anatomic landmarks from the planning CTA, in order to approach the origin of the PA. 3) Distal advancement of the microcatheter/microguidewire or (preferably) advancement of the 5Fr. angiographic catheter over the hydrophilic guidewire in the anterior division of IIA, far from the origins of superior and inferior gluteals and closer to the expected PA origin. Anteroposterior angiograms performed from this distal position depicted the target vessels without superposition of the muscular (superior/inferior gluteal) branches and facilitated identification of the PA. 4) Gentle probing with microcatheter and microguidewire at the expected site of origin of the PA. A common combination was a microcatheter with angled tip (Direxion 2.4Fr, Bern shape, Boston Scientific) and a double-angled 0.016”microguidewire (Meister- Asahi Intecc co, Japan).

No more than 3–5 min of operation time and approximately 1 min of fluoroscopy per pelvic side were devoted to these attempts. If they proved fruitless, steep oblique DSAs were acquired (25-45^o^ with additional caudal-cranial angulation 8-12^o^,taking into account the angle of oblique MIPs that optimized detection of PA origin), as per standard PAE technique (S-PAE).

Prostatic arteries were subsequently catheterized with the microcatheter and microguidewire; nitroglycerin (250 μg) was administered through the microcatheter for vasodilation and additional angiograms (only anteroposterior, in case of AP-PAE) were performed with manual injection of 2-4 ml of contrast through the microcatheter. Embolization was started at the (preferably distal) extraprostatic part of the PA, after advancement of the microcatheter beyond the potential origin of collateral branches to the bladder, rectum or penis. When complete flow stasis was observed, advancement of the microguidewire (followed by the microcatheter) into the intraprostatic branches was attempted (“PErFecTED” technique: “Proximal Embolization First, Then Embolize Distal”, Carnevale et al. 2014).PAE was performed with microspheres (Embozene- Boston Scientific, or Embosphere-Merit Medical) with diameters of 100–500 μm. Prerequisites for selection of smaller microspheres (Embozene 250, or Embosphere 100–300) were: i) Advancement of the microcatheter at the distal extraprostatic part of the PA, or into the prostate. ii) Absence of large intraprostatic anastomoses with penile or rectal branches. iii) Safe distance of the microcatheter tip from the origins of vesical, penile or rectal arteries. All other cases were treated with Embozene 400, or Embosphere 300–500 microspheres. Selection of the one manufacturer over the other depended on the material availability at the time of intervention.

For all PAE procedures the same angiographic protocol of the equipment (‘General DSA’) was selected. All DSA acquisitions were performed at 1frame/second (fr/s) and fluoroscopy at 7.5 fr/s. Additional exposure parameters are provided in Table 2 of supplementary material. A Cone-Beam Computed Tomography (CBCT) facility was not available during the study period. Intraprocedural contrast-enhanced ultrasonography (CEUS), with a second-generation echo enhancer (SonoVue, Bracco, Milan, Italy), with a portable unit (M8 Mindray, Nanshan, Shenzhen, China) was utilized to depict the area fed by the catheterized vessel and to evaluate the ischemic effect immediately post PAE (Moschouris et al. 2020). All procedures were performed by two interventional radiologists, each with 2 years of previous experience in PAE and more than 12 years in other pelvic embolization procedures. Postoperative care was similar to previous work (Moschouris et al. 2019). In case of transradial access, patients were discharged 5–6 h post PAE.

### Evaluation of procedural parameters, efficacy and safety

After each PAE procedure, several parameters (fluoroscopy time, dose-area product-DAP, number of acquisitions with the respective C-arm projections, field size, irradiation parameters) were recorded by the angiographic unit and were extracted for analysis.

For the purposes of this study, a limited, short-term evaluation of imaging and clinical outcomes was performed. Transabdominal CEUS was performed in all patients 18–24 h post PAE to measure the extent of prostatic infarction. Transabdominal US was performed 3 months post PAE and changes (compared to baseline measurements) in prostate volume and post-void residual were recorded Further details on sonographic intra- and postprocedural monitoring of embolization are provided in Table 3 of supplementary material. The International Prostate Symptom Score (IPSS) was also calculated 3 months post PAE and compared with baseline. Clinical success was defined as an IPSS≤15 points with a decrease of at least 25% from the baseline and with no need for additional medical or surgical treatment post PAE. For patients with IBC, clinical success was defined as permanent catheter removal with spontaneous micturition and post void residual (PVR) < 100 ml. In these patients, trials of catheter removal were performed every week for the first month post PAE and every 2 weeks for the second month.

### Comparisons and statistical analysis

Patients who underwent bilateral embolization, with AP-PAE of at least one pelvic side formed the AP-PAE group; patients who underwent bilateral embolization with standard technique (first with anteroposterior *and then* with oblique imaging of *both* pelvic sides) formed the S-PAE group. Descriptive statistics were calculated for quantitative and qualitative data for both groups. Differences in baseline characteristics, procedural, technical parameters and outcomes between the two groups were evaluated. Particularly for the S-PAE group, comparable anteroposterior and oblique angiographic runs (of the same anatomic area and with the same frame number and collimation) were identified and the respective DAPs were recorded. Several tests were used for comparisons, depending on the kind, sample size and distribution of each variable (t-test, Welch test, Mann-Whitney U test, Chi-Square test). Statistical significance was defined as a *p* value of < 0.05.

## Results

Forty-six patients (92 pelvic sides) were studied. AP-PAE group included 12/46 patients (26%). Unilateral AP-PAE could be achieved in 9/12 patients (19.5% of all patients of the study). In unilateral AP-PAE, the contralateral pelvic side was embolized after additional oblique imaging, as per standard technique. Bilateral AP-PAE was performed in 3/12 patients (6.5% of all patients of the study), resulting in a total of 15/92 (16.3%) pelvic sides which were embolized with utilization of anteroposterior imaging only (Figs 1, 2 of supplementary material). No cases of dissection or other iatrogenic vascular injury were observed during the manoeuvres of AP-PAE.

### Anatomic features of the two groups

Compared to the S-PAE group (34/46 patients), AP-PAE group had significantly larger prostates (*p* = 0.047) and larger prostatic arteries (*p* < 0.001). Other baseline demographic, clinical and most of the anatomic features were comparable between the two groups (Table 1).

**Table 1:** Baseline demographic, anatomic and clinical data for the two patient groups of this study

	S-PAE (*n* = 34)	AP-PAE (*n* = 12)	*P*-value
Age (yrs), (mean ± SD, years)	73.3 ± 7.9	73.9 ± 9.7	0.832
BMI, (mean ± SD)	27.0 ± 3.5	27.9 ± 3.6	0.451
PV (mean ± SD, ml)	89.5 ± 27.4	111.4 ± 42.8	0.047*
PA diameter (mean ± SD, mm)	1.4 ± 0.2	1.7 ± 0.2	< 0.001*
Tortuosity Index (mean ± SD)	2.3 ± 0.8	1.9 ± 0.8	0.152
Stenosis index (mean ± SD)	1 ± 0.42	1 ± 0.94	1.000
LUTS, proportion of pts	25/34	9/12	0.921
IPSS (mean ± SD)	25.5 ± 5.6	28.8 ± 3.3	0.107
PVR (mean ± SD, ml)	158 ± 62	143 ± 87	0.580
IBC (% of pts)	26.5 (*n* = 9)	25 (*n* = 3)	0.921

*SD* standard deviation, *BMI* body mass index, *PV* prostate volume, *PA* prostate artery, *LUTS* lower urinary tract symptoms, *IPSS* international prostate symptom score, *PVR* post void residual, *IBC* indwelling bladder catheter

*statistically significant

Regarding the origin of PA, type IV (prostatic artery origin from the internal pudendal) was far commoner among the pelvic sides treated with AP-PAE (6/15 pelvic sides or 40%, versus 15/77 or 19.5% of the pelvic sides treated with S-PAE, *p* = 0.085). On the contrary, type I (common origin of the superior vesical and inferior vesical-prostatic artery) was far commoner among the pelvic sides treated with S-PAE (34/77 pelvic sides or 44.1%, versus 3/15 or 20% of the pelvic sides treated with AP-PAE, *p* = 0.082). The prevalence of type III (prostatic artery origin from the obturator artery) was slightly higher among pelvic sides of AP-PAE and type II (prostatic artery origin from the anterior division of the internal iliac independent from and inferior to the superior vesical) was slightly more common among pelvic sides of S-PAE (Table 4 of supplementary material). In one of the 15 pelvic sides treated with AP-PAE, PA originated from accessory pudendal artery (type V). We also observed 2 type V origins in the 77 pelvic sides treated with standard technique (from accessory pudendal artery, *n* = 1, and from trifurcation of anterior division, *n* = 1). There was one case of AP-PAE, in which planning CTA indicated PAE duplication, but only one of the 2 PAs could be identified and catheterized during the procedure. On the other hand, during S-PAE, 2 cases of PA duplication were correctly recognized and appropriately managed. Two cases of anastomoses of PA with middle rectal artery (MRA) could be identified during AP-PAE; in both, it was possible to advance the microcatheter distal to the origin of the MRA.

### Radiation and other procedural data

Mean fluoroscopy time was 20% shorter in AP-PAE group, although the difference did not reach statistical significance (*p* = 0.084). However, the mean DAP of AP-PAE was 35.9% lower compared to S-PAE (22,924.9 vs 35,800.4 μGy^.^m^2^) and this difference was statistically significant (*p* = 0.018, Table 2). In the S-PAE group, 52 pairs of comparable anteroposterior and steep oblique angiographic runs were identified. The latter were associated with a significantly higher mean DAP (1255.7 ± 822.7 μGy^.^m^2^ vs 683.7 ± 479.4 μGy^.^m^2^, mean difference: 94.2+/− 76.4%, *p* = 0.001). Additional technical procedural details are provided in Table 5 of supplementary material.

**Table 2:** Comparison of radiation dose - related features of PAE for the two groups

	S-PAE (*n* = 34)	AP-PAE (*n* = 12)	*P*-value
Fluoroscopy time (mean ± SD, min)	57.9 ± 21.7	46.3 ± 11.1	0.084
DAP (mean ± SD,μGy^.^m^2^)	35,800.4 ± 16,819.9	22,924.9 ± 11,305.9	0.018*
Number of runs per session	23.6 ± 6.5	20.3 ± 7.4	0.188
Number of images per session	240.7 ± 66,3	217.2 ± 79.1	0.370
Contribution of acquisitions vs fluoroscopy in DAP (%)	60.6/39.4	52.1/47.9	0.226

*SD* standard deviation, *DAP* dose area product

* statistically significant

### Outcomes and follow up

On early (1 day post PAE) CEUS evaluation, the two approaches resulted in comparable extent of prostatic infarction. Three months post PAE, clinical success rates were 91.6% (11/12 patients) versus 91.1% (31/34 patients) for AP-PAE and for S-PAE group, respectively (*p* = 0.959). The rates of international prostate symptom score (IPSS) reduction and of PV and PVR reduction were also similar for the two groups (Table 3). Only minor complications were observed in 5/34 patients of the S-PAE group (acute urinary retention: *n* = 3, small groin hematoma: *n* = 2) and in 3/12 patients of the AP-PAE group (acute urinary retention: *n* = 1, small forearm hematoma post transradial access: *n* = 1, transient, self-limiting hemospermia: *n* = 1). Differences in prevalence of complications between the 2 groups were not significant (*p* = 0.440).

**Table 3:** Comparison of outcome parameters for the two groups

	S-PAE (*n* = 34)	AP-PAE (*n* = 12)	*P*-value
Percentage of prostatic infarction ^a^ (mean ± SD,%)	32.2 ± 20.7	28.0 ± 11.7	0.510
PV reduction ^b^ (mean ± SD,%)	26.4 ± 15.4	33.0 ± 7.6	0.062
IPSS reduction ^b^ (mean ± SD,%)	58.1 ± 15.9	60.2 ± 16.0	0.740
PVR reduction ^b^ (mean ± SD,%)	65 ± 42	56 ± 50	0.638
Clinical success ratio ^b^ (%)	91.1 (*n* = 3)	91.6 (*n* = 1)	0.959
Complications ^c^ -proportion of pts	5/34	3/12	0.423

*SD* standard deviation, *PV* prostate volume, *IPSS* international prostate symptom score, *PVR* post void residual

^a^calculated 1 day post PAE

^b^calculated 3 months post PAE

^c^only minor complications were observed

## Discussion

This study showed that the AP-PAE approach was associated with a reduction of oblique fluoroscopic and angiographic projections in a relatively small but not negligible percentage (26%) of patients. AP-PAE required only moderate experience, no novel techniques and no specialized equipment. In the short-term, imaging and clinical outcomes of this approach were satisfactory and comparable to those of S-PAE.

Several factors may have contributed to technical success of AP-PAE:

Pelvic CTA has an established role in facilitating planning of original PAE (Bilhim et al. 2012a, 2012b) and it also proved indispensable in the context of AP-PAE. Coronal MIP reconstructions were particularly useful to identify the origin of PA preoperatively and to guide the attempts for catheterization of the PA (or, at least, for catheterization of its parent vessel).

Additionally, the herein reported results are largely attributed to some favorable anatomic aspects of the AP-PAE group. These patients had significantly larger prostates and larger diameters of the PAs and these two features are considered as predictors of technical success of PAE (Enderlein et al. 2020; Hacking et al. 2019; Lintin et al. 2020). Large prostates are fed by large prostatic arteries, which are easier to identify (even when imaging is limited to anteroposterior views) and catheterize. This results in shorter fluoroscopy times, (perhaps more importantly) reduced need for oblique views and, eventually, reduced radiation dose to patient and operator. On the contrary, tortuosity of the pelvic arteries has negative effect on technical outcomes of PAE (Lintin et al. 2020), although for this predictor, the difference in favor of AP-PAE group was not statistically significant.

It is also likely, that technical success of AP-PAE (and the subsequent benefit in terms of reduced dose). depended on the type of origin of PA. Types III and (particularly) IV were more common in AP-PAE than in S-PAE. It was relatively easy to catheterize the obturator or internal pudendal without resorting to oblique views; on subsequent DSA, it was also relatively straightforward to identify the origin of PA from the respective arteries. On the contrary, greater difficulties were experienced in identifying and selecting type I and II origin of PA, and the prevalence of these types was comparatively lower in AP-PAE group. In the context of PAE with the original technique, other researchers (Enderlein et al. 2020; Eldem et al. 2020) have also found a positive effect of type III and IV origins on technical success and on shorter procedure time, whereas type I was a predictor of technical failure of bilateral PAE (Bilhim et al. 2013). Finally, one of the cases with rarer type of PA origin (type V) as well as duplicate PAs could be treated only with S-PAE. This probably indicates that complex and less frequent variants of PA anatomy may not be suitable for AP-PAE.

The present study showed that AP-PAE was associated with a significantly lower (35.9%) mean DAP compared to S-PAE, and this is probably the most important advantage of the former approach. DAP is defined as the incident dose multiplied by the surface area irradiated; DAP represents the entire amount of energy delivered to the patient by the beam and is used as an indirect measure for assessing radiation risk, particularly for stochastic effects (Zumstein et al. 2020; Andrade et al. 2017; Hertault et al. 2015). Taking into account that other determinants of DAP (such as the BMI, operator, equipment and technical parameters) were the same (or not significantly different) between the two groups, the reduced radiation dose in AP-PAE should be attributed to the limited utilization of steep oblique fluoroscopic and angiographic projections.

Since PAE often requires long fluoroscopy times and numerous DSA runs at several projections, concerns have been raised about radiation safety of this procedure. In an elaborate study (Andrade et al. 2017) of several (direct and indirect) radiation parameters, PAE was associated with a greater peak skin dose to the patient (2420.3 mGy) than other complex endovascular procedures. The average effective dose per procedure for the interventional radiologist (17 μSv) was also comparable with other high-exposure interventions. Oblique projections were considered a major cause for the high dose measurements; in line with these observations, the present study showed that in the S-PAE group, the mean DAP of steep oblique angiographic runs was almost twice as high as the mean DAP of comparable anteroposterior runs. Oblique projections are associated with increased scatter radiation, as a result of the increased amount of tissue that needs to be traversed by the X-rays. Of note, an exponential increase of scattered radiation is observed when steep (> 30^o^) right or left oblique views are applied (Hertault et al. 2015). Radiation exposure of the staff is further increased by the proximity of the X-ray tube to the operator, with left anterior obliquity resulting in higher exposure for an operator standing at the right side of the patient and vice-versa (Sukupova et al. 2018). Finally, steep oblique views may interfere with correct arrangement of the movable lead shields. Garzón et al. (2016) found that the combination of left anterior oblique projections and inappropriate positioning of ceiling suspended lead shield during PAE could result in high radiation doses to the eyes of the operator. If utilization of lead glasses had also been neglected, the annual equivalent dose limit for the eyes would have been reached with just one PAE procedure per week.

Taken together, all these data emphasize the need for careful and systematic application of the ALARA principle in the clinical context of PAE; strict adherence to the ALARA concept in other complex endovascular procedures has resulted in dramatic reduction of radiation exposure (Hertault et al. 2018). In selected patients, the herein proposed reduction of steep oblique views during PAE appears to be a feasible step towards the implementation of this principle.

A significant methodological limitation of this work should be acknowledged: Anteroposterior imaging was applied at the beginning of the procedure not only to the AP-PAE but also to the S-PAE group. Therefore, the increment in radiation exposure in the latter group could be partially attributed to the fact that S-PAE patients received both anteroposterior and oblique angiographic imaging. However, the initial anteroposterior imaging (applied to both groups) consisted only of a minute of fluoroscopy and one run per pelvic side, so its contribution to the overall radiation exposure of the S-PAE group was small. Moreover, several operators utilize both anteroposterior and oblique projections in their standard PAE practice.

Additional limitations of the present study should also be mentioned: PAE was performed with older equipment, and the potential benefits of CBCT could not be evaluated, as this technique was not available. This was a retrospective study with small groups and short follow-up time. Only indirect measures of radiation dose were recorded, which are less accurate than direct ones (such as peak skin dose). Very few patients with significant pelvic arterial stenoses and no patients treated with unilateral PAE were studied, therefore AP-PAE was not tested in more technically challenging cases. For the same reason, the high clinical success rates reported in this study do not accurately reflect the clinical efficacy of AP-PAE under real-life conditions.

## Conclusions

AP-PAE appears to be a feasible way to significantly reduce radiation exposure during PAE, with no compromise on safety and efficacy. However, the technique can be applied in a relatively small percentage of patients.

## Supplementary Information

**Additional file 1: Table 1**. Basic parameters of pelvic CTA utilized for this study. **Table 2**. Exposure and other technical parameters of DSA. **Table 3**. Basic features of the US modalities which were applied for evaluation of PAE. **Table 4**. Prevalence of different types of origin of PA for the two groups. **Table 5**. Comparison of technical features of PAE for the two groups. **Figure 1**. Application of AP-PAE in a case of type III origin of the right PA. Oblique sagittal (A), and anteroposterior (B), MIP reconstructions from the planning CTA show the obturator artery (open arrows) and the PA (arrows) originating from the proximal part of the obturator. Anteroposterior road-map image of the right IIA (C) and of its branches also shows the obturator artery (open arrow) and the PA (arrow), although the origin of the latter cannot be clearly identified. The obturator could be easily catheterized and selective angiography (D), with the microcatheter at its proximal part (open arrow), clearly showed the origin of the PA (arrow). Subsequent catheterization of the PA was also relatively easy (selective PA angiogram, E). Intraprocedural CEUS (F), after IV injection of SonoVue immediately post embolization of right PA, shows extensive devascularization of the right hemiprostate (asterisks). **Figure 2**. Application of AP-PAE in a case of bilateral type IV PA origin. Anteroposterior MIP reconstruction (A), from the planning CTA, shows PA of both sides (arrows) originating from the internal pudendals. Anteroposterior road-map image of the left IIA and of its branches (B), shows advancement of the microcatheter at the middle third of the internal pudendal (arrow). It was easy to proximally retract the microcatheter and then select the PA. Anteroposterior angiograms (C,D) show the tip of the microcatheter (arrows) in the extraprostatic part of the left PA. Anteroposterior angiogram of the right IIA and of its branches (E), shows the origin of the right PA (open arrow) from the internal pudendal. Anteroposterior angiograms with the tip of the microcatheter at the origin and more distally in the right PA (arrows at F and G, respectively) show the typical appearance of the PA. Intraprocedural CEUS (H), after IV injection of SonoVue at the end of the procedure, shows extensive devascularization of both prostatic lobes (asterisks)

## Data Availability

The datasets used and/or analysed during the current study are available from the corresponding author on reasonable request.

## Abbreviations

AP: Anteroposterior
BMI: Body mass index
CEUS: Contrast-enhanced ultrasonography
CTA: Computed tomographic angiography
DSA: Digital subtraction angiography
IBC: Indwelling bladder catheter
IIA: Internal iliac artery
IPSS: International prostate symptom score
IV: Intravenous
LUTS: Lower urinary tract symptoms
MIP: Maximum intensity projection
MRA: Middle rectal artery
PAE: Prostatic artery embolization
PA: Prostatic artery
PV: Prostate volume
PVR: Post void residual
S: Standard
US: Ultrasonography
