# Supplementary material for: Prostatic artery embolization performed in anteroposterior projections versus steep oblique projections: single centre retrospective comparative analysis
Source: CVIR Endovasc. 2021 Feb 9;4:21. doi: 10.1186/s42155-021-00209-7 (PMC7873151; doi:10.1186/s42155-021-00209-7)
Supplement: Supplementary file 1 — Additional file 1: Table 1. Basic parameters of pelvic CTA utilized for this study. Table 2. Exposure and other technical parameters of DSA. Table 3. Basic features of the US modalities which were applied for evaluation of PAE. Table 4. Prevalence of different types of origin of PA for the two groups. Table 5. Comparison of technical features of PAE for the two groups. Figure 1. Application of AP-PAE in a case of type III origin of the right PA. Oblique sagittal (A), and anteroposterior (B), MIP reconstructions from the planning CTA show the obturator artery (open arrows) and the PA (arrows) originating from the proximal part of the obturator. Anteroposterior road-map image of the right IIA (C) and of its branches also shows the obturator artery (open arrow) and the PA (arrow), although the origin of the latter cannot be clearly identified. The obturator could be easily catheterized and selective angiography (D), with the microcatheter at its proximal part (open arrow), clearly showed the origin of the PA (arrow). Subsequent catheterization of the PA was also relatively easy (selective PA angiogram, E). Intraprocedural CEUS (F), after IV injection of SonoVue immediately post embolization of right PA, shows extensive devascularization of the right hemiprostate (asterisks). Figure 2. Application of AP-PAE in a case of bilateral type IV PA origin. Anteroposterior MIP reconstruction (A), from the planning CTA, shows PA of both sides (arrows) originating from the internal pudendals. Anteroposterior road-map image of the left IIA and of its branches (B), shows advancement of the microcatheter at the middle third of the internal pudendal (arrow). It was easy to proximally retract the microcatheter and then select the PA. Anteroposterior angiograms (C,D) show the tip of the microcatheter (arrows) in the extraprostatic part of the left PA. Anteroposterior angiogram of the right IIA and of its branches (E), shows the origin of the right PA (open arrow) from the internal puden [file 42155_2021_209_MOESM1_ESM.docx]

**Table 1. Basic parameters of pelvic CTA utilized for this study**

| Equipment | 64-row scanner (Optima CT 660, GE Healthcare, WI, USA) |
| --- | --- |
| Kilovoltage | 120 kV |
| Milliamperage | Automatic |
| Matrix | 512x512 pixels |
| Collimation | 64x1.25 mm |
| Slice thickness | 1.25 mm |
| Pitch | 0.984 |
| Iodinated contrast | 150 ml, 350 mg I/ml |
| I.V. Injection rate | 4.5-5 ml/s |
| Bolus triggering | With ROI just above aortic bifurcation, threshold of 250 HU |

CTA computed tomographic angiography; IV intravenous; ROI region of interest; HU Hounsfield units

**Table 2.** **Exposure and other technical parameters of DSA**

| Angiographic protocol | “General DSA” |
| --- | --- |
| Rate of DSA acquisitions | 1frame/sec |
| Rate of fluoroscopy | 7.5frames/sec |
| Kilovoltage (DSA) | 61-106 kVp |
| Milliamperage (DSA) | 444-699 mA |
| Pulse width (DSA) | 69-125 ms |
| Added filtration | 0.0-0.1 mm Copper |
| Focus size | Small in approx. 85% of acquisitions |

**Table 3. Basic features of the US modalities which were applied for evaluation of PAE**

| Modality | Technique | Findings and measurements |
| --- | --- | --- |
| Baseline US | Scanning of prostate and bladder at 3 planes | Assessment of prostate size and shape. Calculation of baseline PV and PVR (ellipsoid formula) |
| Baseline CEUS | Bolus IV injection of 2^nd^ generation US contrast agent (1,5-2ml of SonoVue, Bracco Imaging, Milan, Italy). Contrast specific, real-time, continuous scanning, low mechanical index (MI=0.12-0.13) technique | Assessment of prostate enhancement (almost invariably strong and homogeneous enhancement in the cases of BPH included in this study) |
| Intraprocedural CEUS-IA | IA administration of diluted US contrast agent. Scanning of the prostate and surrounding organs | Rapid, intense enhancement of the targeted prostatic lobe, if PA had been correctly selected; otherwise, enhancement of neighbouring organs |
| Intraprocedural CEUS-IV | Same with baseline CEUS | Reduced prostatic enhancement, non-enhancing infarcts |
| Postprocedural US | Same with baseline US | Same with baseline US |
| Postprocedural CEUS | Same with baseline CEUS | Reduced prostatic enhancement, non-enhancing infarcts.  Measurement of infarcts at 3 planes, calculation of infarct volume with ellipsoid formula, calculation of percentage of prostatic infarction: (Infarct Volume/PV)x100% |
|  |  |  |

US ultrasound; CEUS contrast-enhanced ultrasound; PV prostate volume; PVR post void residual volume; IV intravenous ; IA intraarterial

**Table 4. Prevalence of different types of origin of PA for the two groups**

| Type of PA origin | S-PAE  (n=77 pelvic sides) | AP-PAE  (n=15 pelvic sides) | P value |
| --- | --- | --- | --- |
| I (% of pelvic sides) | 44.1 (n=34) | 20 (n=3) | 0.082 |
| II (% of pelvic sides) | 19.5 (n=15) | 13.3 (n=2) | 0.576 |
| III (% of pelvic sides) | 14.3 (n=11) | 20 (n=3) | 0.575 |
| IV (% of pelvic sides) | 19.5 (n=15) | 40 (n=6) | 0.085 |
| V (% of pelvic sides) | 2.6 (n=2) | 6.6 (n=1) | 0.417 |

**Table 5. Comparison of technical features of PAE for the two groups**

|  | S-PAE (n=34) | AP-PAE (n=12) | P-value |
| --- | --- | --- | --- |
| Arterial access *transfemoral/transradial* | 4/30 | 4/8 | 0.093 |
| Angiographic catheter *reverse curve or double angle /single angle* | 25/9 | 4/8 | 0.014* |
| “PErFecTED” technique proportion of pts | 16/34 | 3/12 | 0.186 |
| Diameter of microspheres used for embo- proportion of pts |  |  |  |
| - *<=300 microns* | 20/34 | 6/12 | 0.600 |
| - *300-500 microns* | 10/34 | 2/12 | 0.392 |
| - *both* | 4/34 | 4/12 | 0.093 |

*In the majority of our cases, AP-PAE could be achieved with single angled catheters (vertebral or multipurpose). We speculate that the single, wide angle of these catheters allows for a better alignment of the catheter with the proximal part of the internal iliac artery and facilitates distal advancement of the catheter in the anterior division of the internal iliac artery. Additionally, the aforementioned simple catheter shapes probably provide increased torquability and pushability of the coaxial microcatheter compared to catheters with double angle or reverse curve.

Fig. 1


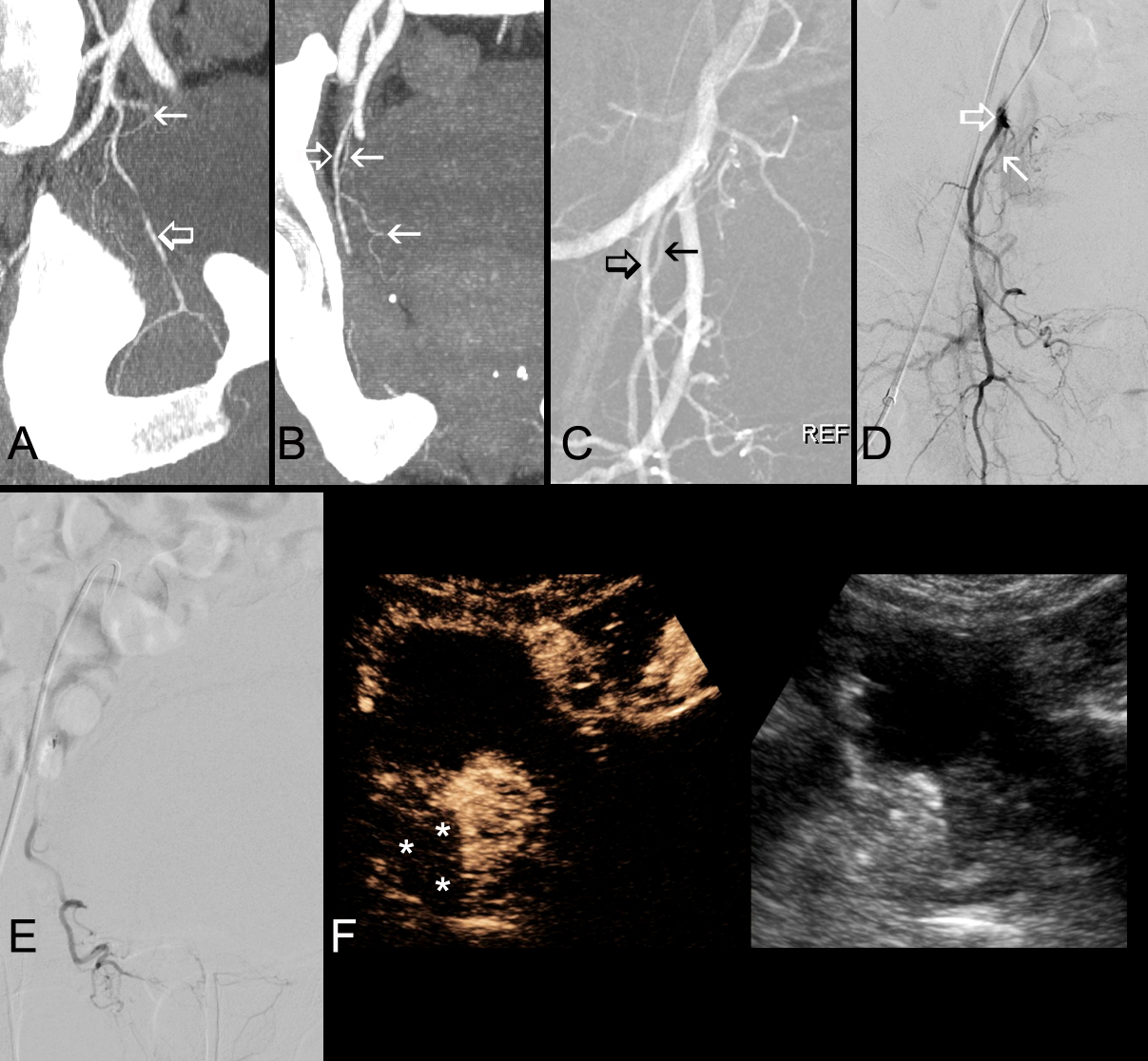


Application of AP-PAE in a case of type III origin of the right PA. Oblique sagittal (A), and anteroposterior (B), MIP reconstructions from the planning CTA show the obturator artery (open arrows) and the PA (arrows) originating from the proximal part of the obturator. Anteroposterior road-map image of the right IIA (C) and of its branches also shows the obturator artery (open arrow) and the PA (arrow), although the origin of the latter cannot be clearly identified. The obturator could be easily catheterized and selective angiography (D), with the microcatheter at its proximal part (open arrow), clearly showed the origin of the PA (arrow). Subsequent catheterization of the PA was also relatively easy (selective PA angiogram, E). Intraprocedural CEUS (F), after IV injection of SonoVue immediately post embolization of right PA, shows extensive devascularization of the right hemiprostate (asterisks).

Fig. 2


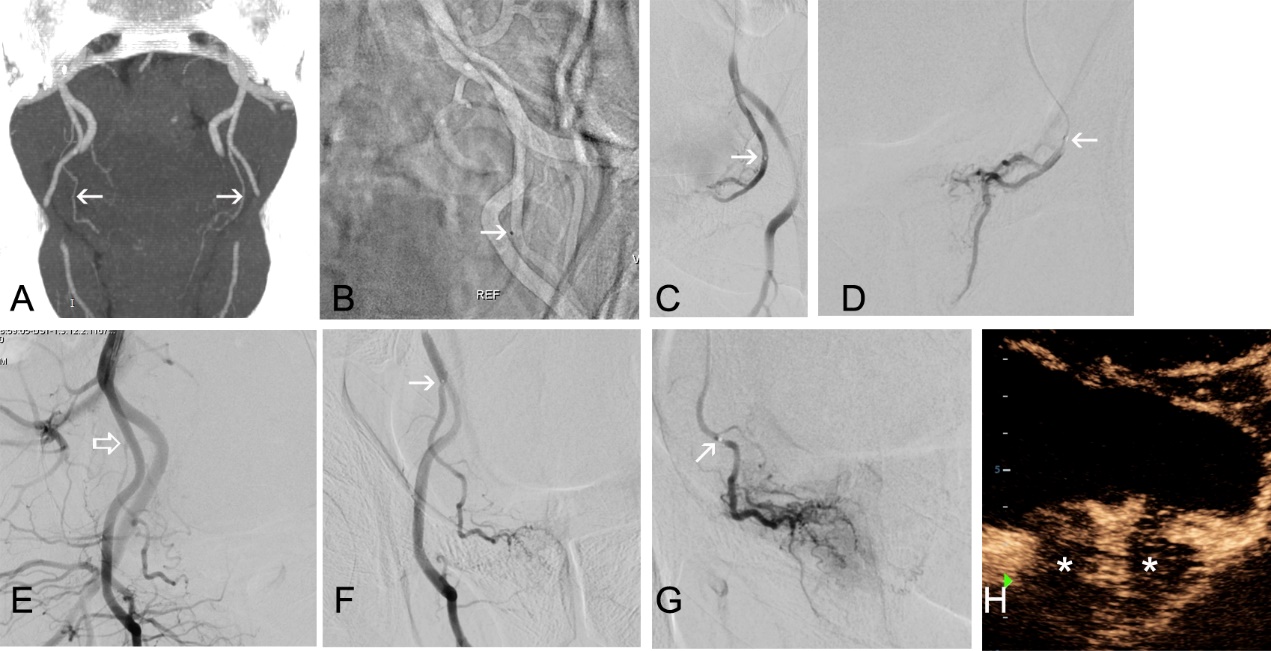


Application of AP-PAE in a case of bilateral type IV PA origin. Anteroposterior MIP reconstruction (A), from the planning CTA, shows PA of both sides (arrows) originating from the internal pudendals. Anteroposterior road-map image of the left IIA and of its branches (B), shows advancement of the microcatheter at the middle third of the internal pudendal (arrow). It was easy to proximally retract the microcatheter and then select the PA. Anteroposterior angiograms (C,D) show the tip of the microcatheter (arrows) in the extraprostatic part of the left PA. Anteroposterior angiogram of the right IIA and of its branches (E), shows the origin of the right PA (open arrow) from the internal pudendal. Anteroposterior angiograms with the tip of the microcatheter at the origin and more distally in the right PA (arrows at F and G, respectively) show the typical appearance of the PA. Intraprocedural CEUS (H), after IV injection of SonoVue at the end of the procedure, shows extensive devascularization of both prostatic lobes (asterisks).
